# A Real-World Prospective Cohort Study of Patients With Newly Diagnosed Crohn’s Disease Treated by a Multidisciplinary Team: 1-Year Outcomes

**DOI:** 10.1093/crocol/otad064

**Published:** 2023-10-18

**Authors:** Henit Yanai, Tali Sharar Fischler, Idan Goren, Hagar Eran-Banai, Jacob E Ollech, Yifat Snir, Yelena Broitman, Revital Barkan, Tamar Pfeffer-Gik, Lihi Godny, Yelena Kutokov, Adi Friedeberg, Maor H Pauker, Keren Masha Rabinowitz, Irit Avni-Biron, Iris Dotan

**Affiliations:** IBD Center, Division of Gastroenterology, Rabin Medical Center, Petah Tikva, Israel; Faculty of Medicine, Tel Aviv University, Tel Aviv, Israel; IBD Center, Division of Gastroenterology, Rabin Medical Center, Petah Tikva, Israel; Faculty of Medicine, Tel Aviv University, Tel Aviv, Israel; IBD Center, Division of Gastroenterology, Rabin Medical Center, Petah Tikva, Israel; Faculty of Medicine, Tel Aviv University, Tel Aviv, Israel; Department of Inflammation and Immunity, Lerner Research Institute, Cleveland Clinic, Cleveland, Ohio, USA; IBD Center, Division of Gastroenterology, Rabin Medical Center, Petah Tikva, Israel; Faculty of Medicine, Tel Aviv University, Tel Aviv, Israel; IBD Center, Division of Gastroenterology, Rabin Medical Center, Petah Tikva, Israel; Faculty of Medicine, Tel Aviv University, Tel Aviv, Israel; IBD Center, Division of Gastroenterology, Rabin Medical Center, Petah Tikva, Israel; Faculty of Medicine, Tel Aviv University, Tel Aviv, Israel; IBD Center, Division of Gastroenterology, Rabin Medical Center, Petah Tikva, Israel; Faculty of Medicine, Tel Aviv University, Tel Aviv, Israel; IBD Center, Division of Gastroenterology, Rabin Medical Center, Petah Tikva, Israel; Faculty of Medicine, Tel Aviv University, Tel Aviv, Israel; IBD Center, Division of Gastroenterology, Rabin Medical Center, Petah Tikva, Israel; Faculty of Medicine, Tel Aviv University, Tel Aviv, Israel; IBD Center, Division of Gastroenterology, Rabin Medical Center, Petah Tikva, Israel; Faculty of Medicine, Tel Aviv University, Tel Aviv, Israel; IBD Center, Division of Gastroenterology, Rabin Medical Center, Petah Tikva, Israel; Faculty of Medicine, Tel Aviv University, Tel Aviv, Israel; IBD Center, Division of Gastroenterology, Rabin Medical Center, Petah Tikva, Israel; Faculty of Medicine, Tel Aviv University, Tel Aviv, Israel; IBD Center, Division of Gastroenterology, Rabin Medical Center, Petah Tikva, Israel; Faculty of Medicine, Tel Aviv University, Tel Aviv, Israel; IBD Center, Division of Gastroenterology, Rabin Medical Center, Petah Tikva, Israel; Faculty of Medicine, Tel Aviv University, Tel Aviv, Israel; Felsenstein Medical Research Center, Faculty of Medicine, Tel Aviv, Israel; IBD Center, Division of Gastroenterology, Rabin Medical Center, Petah Tikva, Israel; Faculty of Medicine, Tel Aviv University, Tel Aviv, Israel; IBD Center, Division of Gastroenterology, Rabin Medical Center, Petah Tikva, Israel; Faculty of Medicine, Tel Aviv University, Tel Aviv, Israel

**Keywords:** Crohn’s disease, therapeutic targets, multidisciplinary team, real world

## Abstract

**Background:**

Real-world data on outcomes of patients with newly diagnosed Crohn’s disease (ndCD) is limited. We aimed to assess the achievement of corticosteroid-free clinical remission (CS-free CR) and other therapeutic targets 1 year after diagnosis in a cohort of patients with ndCD treated by a multidisciplinary team (MDT).

**Methods:**

A prospective observational cohort study was conducted on consecutive treatment-naïve adults with ndCD. Patients received management at the treating physician’s discretion, along with a tailored nutritional plan provided by an inflammatory bowel disease (IBD)-oriented dietitian. Patients were guided and educated by an IBD nurse, with flexible communication access to the IBD team. Therapeutic targets were assessed at 1 year. Multivariable logistic regression was used to evaluate predictors of CS-free CR.

**Results:**

Seventy-six patients (50% female) with a median age of 27 (22–39) years were eligible. Over 75% of patients were assessed by IBD-oriented dietitians and the IBD nurse. Within a median of 4.3 (2.5–6.7) months from diagnosis 60.5% initiated biologics (96% anti- tumor necrosis factor). Dietary intervention was applied to 77.6% of the cohort, either monotherapy (33.9%) or add-on (66.1%). At 1 year, 64.5% of patients achieved sustained CS-free CR, 56.6% biochemical remission, 55.8% endoscopic response, 44.2% endoscopic remission, 30.8% deep remission, and in 39.5% there was an improvement in health-related quality of life (HRQoL). Predictors for CS-free CR were uncomplicated phenotype (B1/P0), lower body mass index, and lower patient-reported outcome 2 scores at diagnosis.

**Conclusions:**

In a real-world setting at a tertiary medical center, a cohort of ndCD patients treated by an MDT resulted in favorable 1-year outcomes. Over 60% achieved CS-free CR, along with significant improvements in biomarkers and HRQoL.

## Graphical Abstract

**Graphical Abstract:**
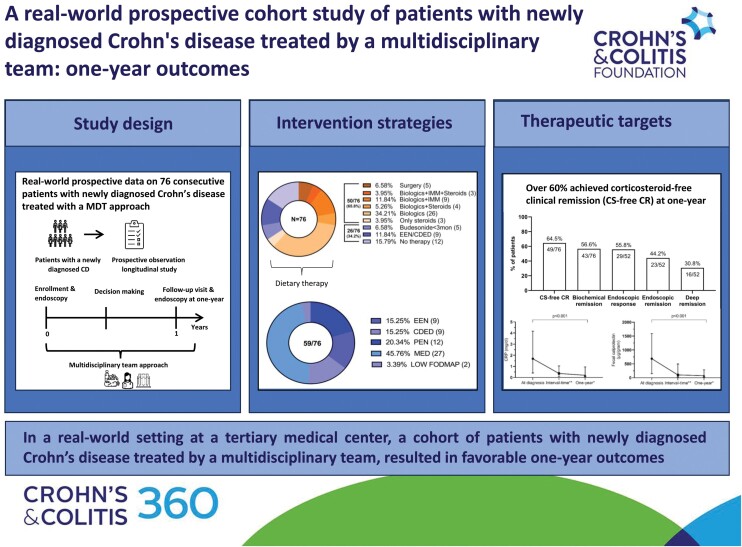

## Introduction

 Crohn’s disease (CD) is a chronic inflammatory bowel disease (IBD) that often has a progressive course. Clinical presentation and treatment response are heterogeneous. Despite the advancement of new therapies and medical strategies, over 50% of patients will develop complications that may lead to surgery over time,^1–3^ and up to 50% of patients experience at least 1 extraintestinal manifestation.^4^ Moreover, CD significantly impairs daily life and psychological well-being and may lead to permanent disability.^5^

During the past decade, top-down, early intervention, tight monitoring, and treat-to-target strategies were introduced to improve clinical outcomes.^6–10^ Implementation of these strategies was associated with reduction in disease complications.^11–13^ The real-world application of such strategies in synchronization requires a multidisciplinary team (MDT) approach.^14^ However, data on the actual impact of MDT application on patient outcomes are scarce.

We have established a structured MDT approach for all patients in our IBD center. Focusing on patients with CD, we recruited and prospectively observed an inception cohort of patients with newly diagnosed CD (ndCD) who are treated and cared for according to current standards^15^ based on the treating physician’s discretion. A structured MDT approach is applied, and comprehensive clinical and exploratory data are collected.

We hereby aimed to assess the rates of the recommended therapeutic targets at 1 year after diagnosis (based on the STRIDE-II consensuses^7^), specifically looking at sustained corticosteroid-free clinical remission (CS-free CR) and evaluating the possible benefits of our MDT approach.

## Methods

### Study Design and Participants

This prospective observational longitudinal study was conducted in a tertiary IBD center (Rabin Medical Center, Beilinson Campus, Petah Tikva, Israel). Our IBD center is part of Clalit Health Services (the largest health maintenance organization [HMO] in Israel, a public health service) that provides care for patients from the affiliated community clinics in our catchment area and hospital-based patients, allowing a heterogeneous population of patients with ndCD. We screened all consecutive adults (aged ≥ 18 years old) with established ndCD (within the 6 months before enrollment) and those with suspected CD. Patients were eligible to this inception cohort only if CD diagnosis was confirmed according to accepted clinical, endoscopic, and imaging criteria,^16^ see Supplementary Methods for detailed inclusion criteria. Recruitment occurred between 2018 and 2021.

Participants in this inception cohort were managed and followed according to the discretion of the treating physician, based on current standards.^15^ Cases were discussed during weekly MDT meetings, including IBD-oriented gastroenterologists, a dedicated radiology expert, a surgical team, an IBD-oriented registered dietitians (in Israel it is required to complete a specific training program in gastroenterology and IBD), an IBD registered nurse, coordinators, and additional experts as relevant to the discussed case(s). Furthermore, patients received guidance from (1) IBD-oriented dietitians who set a nutritional plan based on physician recommendations, symptoms, clinical disease activity, and phenotype, and (2) an IBD nurse who provided personally tailored guidance, information on the disease and medications, and assistance in reaching the therapeutic goals. Patients communicated with the IBD team via email, messages, or phone calls between clinic visits. Finally, each patient underwent scheduled semiannual study visits for comprehensive data collection (see Supplementary Figure 1—study scheme).

For prediction analysis, we included only patients who were naïve to corticosteroids, immunosuppression, or biologic therapy at recruitment and completed 1 year of follow-up under our care. The end of follow-up was determined by the clinic visit at the 1-year period, defined as the latest visit between 9 and 18 months from diagnosis. To assess trends and dynamics of clinical and biomarker status, we captured data at an interim time point, the middle follow-up period for each participant.

### Data Collection and Processing

Data were collected via a dedicated case report form. Clinical characteristics, blood, and stool samples for fecal calprotectin (FC) were collected at baseline and in each study visit. Anti-*Saccharomyces cerevisiae* antibodies (ASCA) IgG and 7 CD-associated polymorphisms that are associated with susceptibility and prognosis were assessed^17^ (Supplementary details on susceptible genes variation are presented in Supplementary Table 1). Disease activity was determined by the Crohn’s disease activity index (CDAI) and patient-reported outcome 2 (PRO-2). Health-related quality of life (HRQoL) was assessed by the short inflammatory bowel disease questionnaire (SIBDQ).^7^

### Outcomes

We evaluated therapeutic targets according to STRIDE-II recommendations^7^ at 1 year after diagnosis. The primary outcome was the 1-year rate of sustained CS-free CR, defined as a combination of CR based on CDAI < 150 without any CS therapy, active perianal disease, treatment escalation, or hospitalization for more than 3 months preceding the analysis—a stringent outcome. Secondary outcomes were: PRO-2 symptom remission—defined as a mean daily score of abdominal pain ≤ 1 and stool frequency ≤ 3; biochemical remission—defined as a combination of C-reactive protein (CRP) < 1 mg/dL and FC < 150 µg/g; endoscopic response—defined as >50% decrease in simple endoscopic score for CD (SES-CD); endoscopic remission—defined as SES-CD < 3; deep remission—defined as a composite of clinical, biochemical, and endoscopic remission and improvement in HRQoL based on the overall change in the SIBDQ, defined as an increase of 9 points.

### Statistical Analysis

Categorical variables were expressed as the frequency or percentage. Continuous variables were evaluated for normal distribution and expressed as the mean and SD or median and interquartile range (IQR). Comparison of categorical variables was performed using the Chi-square test or Fisher’s exact test, and continuous variables were compared using the Mann–Whitney *U* test. We used the nonparametric Wilcoxon rank test to compare paired samples. For analysis of predictors, we utilized a multivariable logistic regression using a backward stepwise method for variable selection (*P*-value > .1 on the Wald test was used for variable removal). All reported *P*-values are 2-sided, *P*-values < .05 were considered significant. Data were analyzed using SPSS (IBM SPSS Statistics, version 28.0, IBM Corp., Armonk, NY, 2021) and R Foundation for Statistical Computing (version 3.3.3, Vienna, Austria) were used for analysis.

### Ethical Consideration

Local and national ethics committees approved the protocols: Rabin Medical Center IRB approval no. 0298-17; IRB approval no. for genetics analysis 0299-17. Written informed consent was obtained from all participants.

## Results

### Participants

**Figure 1.**
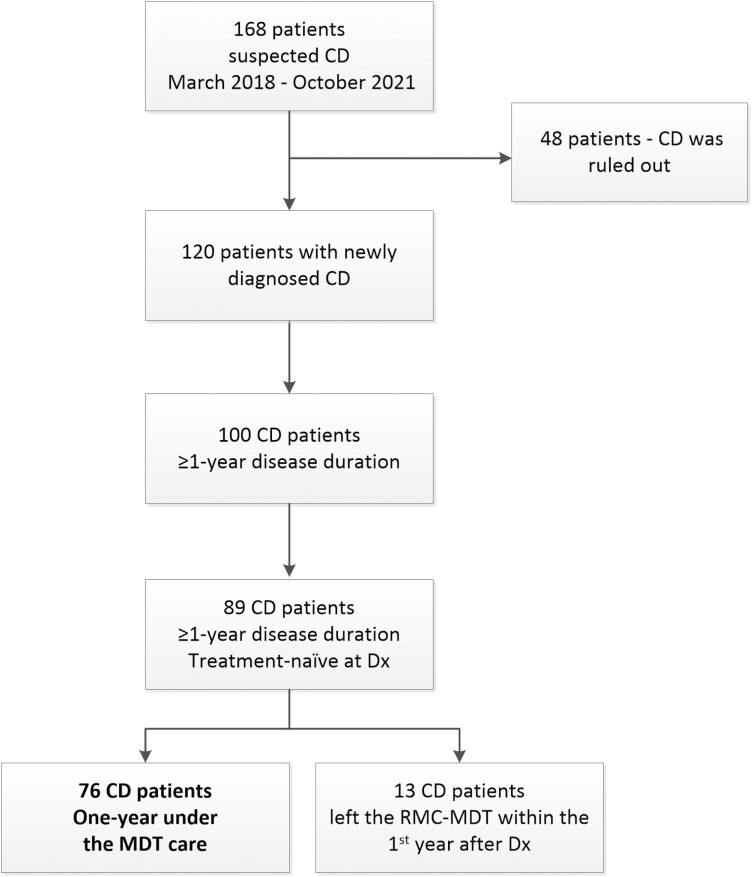
Patient’s disposition. CD, Crohn’s disease; Dx, diagnosis; MDT, multidisciplinary team.

**Figure 2.**
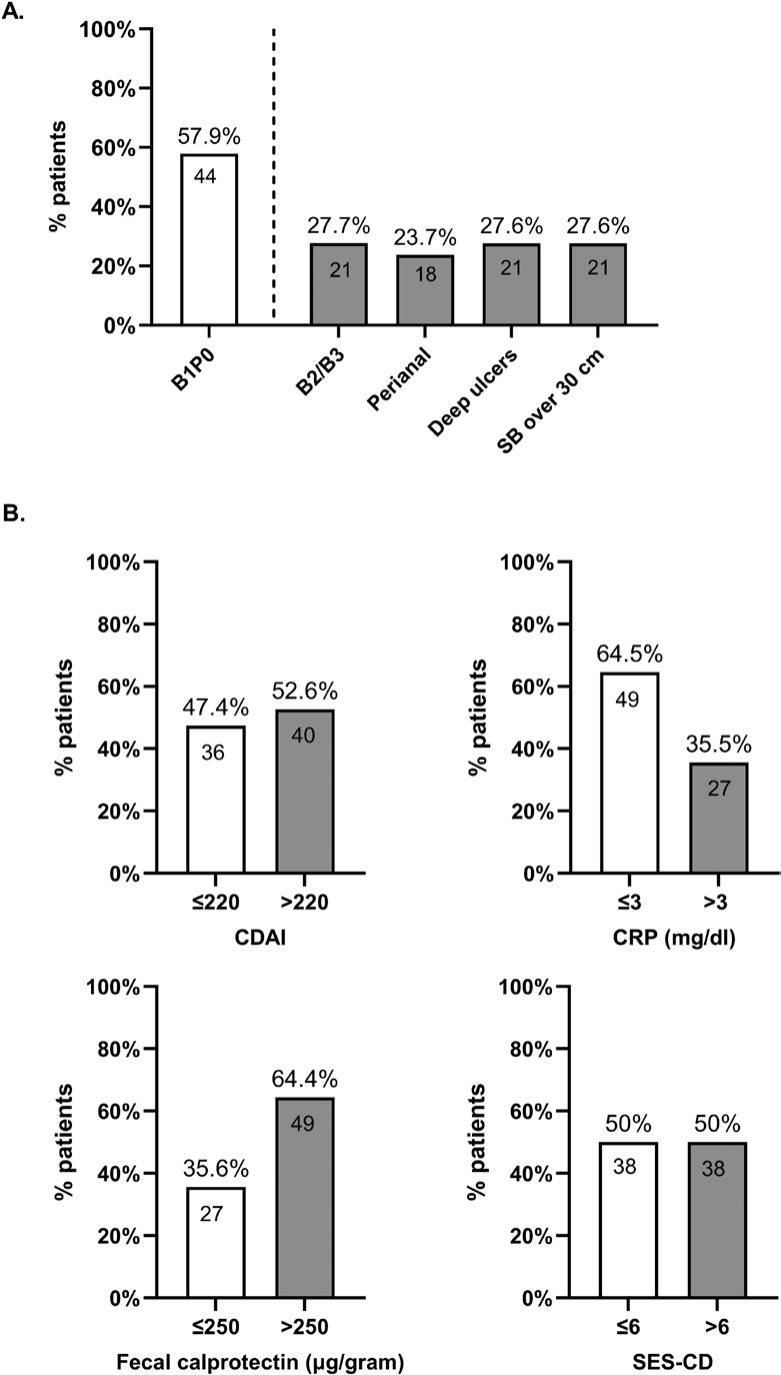
Stratification of the cohort at diagnosis (*N* = 76). (A) Stratification based on disease phenotype at diagnosis: uncomplicated phenotype—inflammatory with no perianal (B1/P0); complicated phenotypes—stricturing or penetrating (B2/B3), perianal disease, deep ulcers, small bowel (SB) involvement over 30 cm. (B) Stratification based on disease activity at diagnosis: CDAI > 220, CRP > 3 mg/dL, fecal calprotectin > 250 µg/g, and SES-CD > 6.

Seventy-six treatment-naïve patients completed 1 year of follow-up under the care of our MDT and were eligible for analysis (Figure 1). Half of the cohort (38/76) were female, the median age at diagnosis was 27 years (IQR: 22–39), 72.4% of patients (55/76) were with an inflammatory phenotype, 23.7% (18/76) with a perianal disease, 52.6% (40/76) had moderate–severe CD (CDAI score > 220) at diagnosis, 35.5% (27/76) had elevated CRP > 3 mg/dL, 64.5% (49/76) had elevated FC > 250 mg/kg, and 50% (38/76) had a SES-CD > 6; see Table 1 patients’ characteristics and Figure 2 for patients stratification according to activity indices.

**Table 1.** Demographics and clinical characteristics at diagnosis (*N* = 76).

Sex, female, *n* (%)	38 (50)
Age, median (IQR)	27 (22–38.7)
BMI (kg/m^2^), median (IQR)	21.7 (20.1–25.3)
Active smokers, *n* (%)	13 (17.1)
Family history of IBD, *n* (%)	20 (26.3)
Origin, Ashkenazi, *n* (%)	38 (50)
Disease location	
Ileal (L1), *n* (%)	47 (61.8)
Colonic (L2), *n* (%)	6 (7.9)
Ileocolonic (L3), *n* (%)	23 (30.3)
Upper GI (L4), *n* (%)	15 (19.7)
Perianal (P), *n* (%)	18 (23.7)
Complex perianal, *n* (%)	9 (11.8)
Disease phenotype	
Inflammatory (B1), *n* (%)	55 (72.4)
Stricturing (B2), *n* (%)	16 (21.1)
Penetrating (B3), *n* (%)	5 (6.6)
Inflammatory without perianal (B1P0), *n* (%)	44 (57.9)
Extraintestinal manifestations, *n* (%)	19 (25)
Symptoms duration till diagnosis (months), median (IQR)	5.5 (3–12)
Disease activity	
Moderate–severe disease (CDAI > 220), *n* (%)	40 (52.6)
CDAI, median (IQR)	223 (131–277)
HBI, median (IQR)	7 (4.2–10)
PRO-2, median (IQR)	6.4 (3.3–13.3)
Stool frequency, median (IQR)	1.7 (0.3–2.8)
Abdominal pain, median (IQR)	1 (0.1–2)
CRP (mg/dL), median (IQR)	1.7 (0.4–1.2)
FC (µg/g), median (IQR)	686 (150–1590)
WBC (10^9^/L), median (IQR)	7.8 (6.3–9.6)
Hemoglobin (g/dL), median (IQR)	13.2 (11.9–14.4)
Albumin (g/dL), median (IQR)	4.5 (4.1–4.8)
SES-CD, median (IQR)	7 (5–11)
Deep ulcers at diagnosis, *n* (%)	21 (27.6)
Small bowel involvement over 30 cm, *n* (%)	21 (27.6)
SIBDQ, median (IQR)	44 (36–55)
** **ASCA positive, *n* (%)	33 (43.4)

Abbreviations: ASCA, anti-*Saccharomyces cerevisiae* antibodies; BMI, body mass index; CDAI, Crohn’s disease activity index; CRP, C-reactive protein; FC, fecal calprotectin; HBI, Harvey–Bradshaw index; IBD, inflammatory bowel disease; PRO-2, patient-reported outcome 2; SES-CD, simple endoscopic score for Crohn’s disease; SIBDQ, short IBD questionnaire; WBC, white blood cell. Values are *n* (%) or median (interquartile range [IQR]).

### Interventions

Within a median time of 1.9 (IQR 1.2–4) months from diagnosis, 71% of patients (54/76) were recommended to start biologic therapy or undergo surgery. In practice, 60.5% (46/76) started a biologic (44/46 anti-tumor necrosis factor [TNF], 1/46 ustekinumab, 1/46 vedolizumab, 2/46 ≥ 1 biologic, within 4.3 months from diagnosis [IQR 2.5–6.7]); and 6.6% (5/76) underwent CD-related surgery (ileocecal resection, *n* = 4; right hemicolectomy, due to a colonic stricture, *n* = 1). All patients treated with a biologic continued therapy until the end of follow-up; 41.3% of patients (19/46) required optimization (13/19 due to partial response, 6/19 due to lose of response, of whom 3 patients with evident immunogenicity). The median duration of biologic therapy at the time of analysis was 10 months (IQR 7–11.25).

In the rest of the cohort (34.2% [26/76 patients]) strategies used for remission induction included: budesonide—6.6% (5/76); dietary monotherapy 11.8% (9/76): 4 patients with exclusive enteral nutrition (EEN) and 5 patients with Crohn’s disease exclusion diet (CDED+/− partial enteral nutrition); and 15.8% (12/76)—expectant management strategy in combination with dietary recommendations. Most of this subgroup (23/26) had an uncomplicated phenotype (B1/P0), of which 16/23 patients with ileal disease (L1), 3/23 with colonic disease (L2), and 4/23 with ileocolonic disease (L3). The 3 other patients included in this subgroup had CD complications that were successfully managed with conservative measures (antibiotics mainly) and declined recommendation to start therapy with a biologic.

Lastly, only 21% (16/76) of our real-world cohort would have been eligible to participate in a registration randomized clinical trial (RCT) if the latest inclusion criteria had been used (CDAI 220–450, SES-CD > 6 or isolated ileal SES-CD 4, and positive PRO-2 criteria). Specifically, patients who required treatment to control CD would have been excluded because of either CDAI > 450 (*n* = 2), CDAI < 220 but elevated inflammatory markers (CRP > 3 mg/dL or FC > 250 µg/g, *n* = 20), low SES-CD but long small bowel disease (*n* = 8), or a complicated perianal disease but low PRO-2 score, low CDAI or a low SES-CD (*n* = 8). All these trial-ineligible patients (*n* = 38/60) were recommended advanced interventions (primarily biologic therapies) to control their disease.

### MDT Management

**Figure 3.**
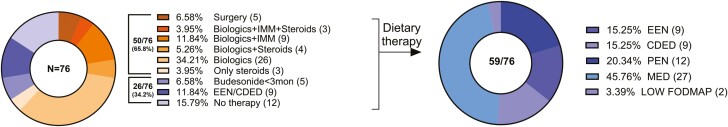
Intervention strategies. (A) Induction strategies. (B) Dietary therapy (monotherapy or add-on therapy). MED, Mediterranean diet recommendation; EEN, exclusive enteral diet; CDED, Crohn’s disease elimination diet; PEN, partial elementary diet; low FODMAP, low fermentable, oligosaccharides, disaccharides, monosaccharides, and polyols. *Indication for dietary add-on therapy—active disease 10/39, stenotic disease 3/39, lifestyle modification 17/39, functional symptoms 1/39, weight gain 8/39.

Overall, 77.6% of patients (59/76) received dietary therapy (either monotherapy—20 patients, or add-on—39 patients) after being assessed by the IBD-oriented dietitian. Intervention strategies are depicted in Figure 3. Following determination of the treatment strategy by the treating physician, 82.9% of patients (63/76) received guidance from an IBD specialist nurse. The nurse met with each of these patients at least once after enrollment to discuss their condition, provide education, and tailor a monitoring and follow-up plan. Most of these patients (90%) maintained real-time contact with the nurse by multiple interactions: repeated frontal visits for guidance (on average 5.3 frontal visits per patient per year), and also phone calls, text messages, or emails as needed (on average 15.7 interactions [any type] per patient per year). Notably, there was a trend toward more IBD nurse interactions with patients with severe characteristics (a complex perianal disease, deep ulcers, extensive small bowel involvement, narrowing or penetrating disease) (*P* = .13). For a detailed number of the different interactions with the MDT, see Supplementary Table 2.

### Therapeutic Targets

**Figure 4.**
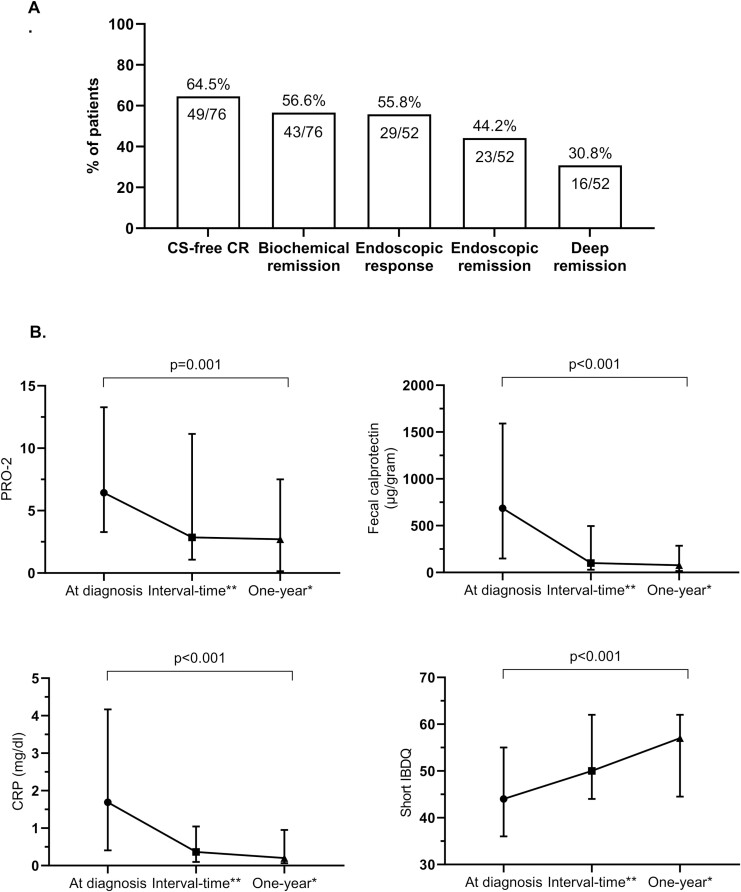
Therapeutic targets. (A) Treatment targets at 1 year after diagnosis: proportion of patients with sustained corticosteroid-free clinical remission (CS-free CR), biochemical remission, endoscopic response, endoscopic remission, and deep remission; endoscopic data were available for 52 patients at 1 year. (B) Alterations in PRO-2 (patient-reported outcome 2), CRP (C-reactive protein), fecal calprotectin, and SIBDQ (short IBD questionnaire) through time—diagnosis to 1 year; the SIBDQ data were available for 73 patients; data represented as median and IQR. * 1-year—latest visit between 9 and 18 months from diagnosis; ** interval time—middle of follow-up period.

At 1 year, 77.6% of patients (59/76) were in CR (CDAI score < 150) and 64.5% (49/76) were in sustained CS-free CR (Figure 4A). Median CDAI decreased from a baseline of 233 (IQR: 131–277) to 73 (IQR: 26–138), *P* < .001. Symptomatic remission by PRO-2 was achieved in 76.7% of patients (56/73), and overall PRO-2 score decreased from 6.4 (IQR: 3.3–13.3) to 2.7 (IQR: 0.14–7.5), both *P* < .001, Figure 4B. Among patients with moderate–severe CD at diagnosis (CDAI score > 220, *n* = 40), there was a similar rate of sustained CS-free CR reaching 65%, and CDAI in this subgroup decreased from 266 (IQR: 244–313) to 84 (IQR: 26–185), *P* < .001. A prespecified sensitivity analysis of a subgroup of patients diagnosed with an uncomplicated phenotype (B1/P0, *n* = 44) revealed 72.7% sustained CS-free CR (comparable between mild–moderate 79.1% [19/24] vs moderate–severe CD 65% [13/20], *P* = .293). Biochemical remission was achieved in 56.6% of patients (43/76), Figure 4A. Conjoined sustained CS-free CR with biochemical remission, with CRP < 0.5 mg/dL, or with FC < 150 μg/g was reached by: 46% (35/76), 61.8% (47/76), and 61.8% (47/76), respectively. The dynamics in biomarkers improvement over time for the entire cohort are depicted in Figure 4B. In a subanalysis for patients who had elevated CRP > 3 mg/dL or FC > 250 μg/g at diagnosis, paired analysis demonstrated a decrease in the median of differences of 4 mg/dL (IQR: 3.1–6.2) and 959 μg/g (IQR: 305–2253), respectively, see Supplementary Figure 2. Paired endoscopic data were available for 52/76 patients, demonstrating endoscopic response, endoscopic remission, and deep remission rates in 55.8%, 44.2%, and 30.8%, respectively. Significant decrease in median SES-CD from 8 (IQR: 4.2–11) to 3 (IQR: 0–5), *P* < .001, was observed over time. HRQoL significantly improved over time in 39.5%, the median SIBDQ score increased from 44 (IQR: 36–55) to 57 (IQR: 44.5–62), Figure 4B.

### Complications and Safety

Within a median of 3 months (IQR: 2.8–11.1), 14.5% of patients (11/76) were hospitalized (disease exacerbation, *n* = 4; small bowel obstruction, *n* = 3; perianal abscess, *n* = 4). Of these, 1 patient had a small bowel obstruction and 4 patients with perianal disease underwent surgery. There were no major treatment adverse events; there was 1 event of mild pancreatitis attributed to 6-mercaptopurine that resolved after drug discontinuation, and 1 minor corneal infection unrelated to the IBD medications.

### Predictors for CS-Free CR at 1-Year

Finally, we assessed whether clinical or biological markers were associated with CS-free CR at 1 year. In univariable analysis, male sex, nonsmoking, lower body mass index (BMI), lower PRO-2 score, and uncomplicated phenotype (B1/P0) at diagnosis were associated with the likelihood of reaching sustained CS-free CR at 1 year (Table 2). Neither ASCA serology nor the genetic variants that are associated with CD course were found to be related with sustained CS-free CR at 1 year. In multivariable regression analysis, only uncomplicated phenotype (B1/P0), lower BMI, and lower PRO-2 score at diagnosis remained significant predictors for sustained CS-free CR (odds ratio [OR] 4.05, 95% CI, 1.24–13.26; *P* = .021, OR 1.15, 95% CI, 1.01–1.30; *P* = .027, and OR 1.1, 95% CI, 1.02–1.18, *P* = .010, respectively), with a receiver operating curve analysis that yielded an area under the curve (AUC) of 0.761 (95% CI, 0.647–0.875). For all other predefined therapeutic targets, no significant predictors were found in this data set.

**Table 2.** Associations with CS-free CR at 1-year, univariate analysis (*N* = 76).

At diagnosis	CS-free CR at 1 year (*n* = 49)	No CS-free CR at 1 year (*n* = 27)	*P*-value
Sex, female, *n* (%)	21 (42.9)	17 (63)	.093
Age, median (IQR)	27 (22–34.5)	27 (24–43)	.252
BMI (kg/m^2^), median (IQR)	21.1 (19.3–24.1)	23 (21.2–27.3)	.021
Active smokers, *n* (%)	7 (14.3)	6 (22.2)	.021
Family history of IBD, *n* (%)	12 (24.5)	8 (29.6)	.626
Origin, Ashkenazi, *n* (%)	25 (51)	13 (48.1)	.811
Disease location			
Ileal (L1), *n* (%)	30 (61.2)	17 (63)	.147
Colonic (L2), *n* (%)	6 (12.2)	0 (0)	
Ileocolonic (L3), *n* (%)	13 (26.5)	10 (37)	
Upper GI (L4), *n* (%)	9 (18.4)	6 (22.2)	.686
Perianal (P), *n* (%)	10 (20.4)	8 (29.6)	.365
Complex perianal, *n* (%)	5 (10.2)	4 (14.8)	.713
Disease phenotype			
Inflammatory (B1), *n* (%)	37 (75.5)	18 (66.7)	.708
Stricturing (B2), *n* (%)	9 (18.4)	7 (25.9)	
Penetrating (B3), *n* (%)	3 (6.1)	2 (7.4)	
Inflammatory without perianal (B1P0), *n* (%)	32 (65.3)	12 (44.4)	.078
Extraintestinal manifestations, *n* (%)	11 (22.4)	8 (29.6)	.489
Symptoms duration till diagnosis (months), median (IQR)	5 (3–12)	6 (3–12.1)	.956
Disease activity			
CDAI, median (IQR)	223 (137–273)	233 (125–282)	.931
HBI, median (IQR)	6 (4–10)	7 (5–9)	.815
PRO-2, median (IQR)	5.1 (2.1–11.6)	10.7 (4.8–19.6)	.019
CRP (mg/dL), median (IQR)	1.5 (0.4–3.7)	3.3 (0.7–4.3)	.365
FC (µg/g), median (IQR)	686 (219–2150)	504 (118–1385)	.246
WBC (10^9^/L), median (IQR)	7.9 (6.3–9.6)	7.8 (6.3–9.9)	.870
Hemoglobin (g/dL), median (IQR)	13.4 (11.9–14.5)	12.9 (11.6–14.4)	.530
Albumin (g/dL), median (IQR)	4.5 (4.1–4.8)	4.5 (4.1–4.8)	.729
SES-CD, median (IQR)	8 (4.5–11)	6 (5–11)	.596
Deep ulcers at diagnosis, *n* (%)	12 (24.5)	7 (25.9)	.890
SIBDQ, median (IQR)	47 (37.5–58)	41 (35–53)	.095
ASCA positive, *n* (%)	21 (43.8)	12 (46.2)	.843
Genetic variants			
NOD2 variant, *n* (%)	2 (3.6)	1 (6.7)	.515
CARD9 variant, *n* (%)	24 (51.1)	13 (54.2)	.804
IRGM variant, *n* (%)	17 (36.2)	13 (54.2)	.146
MHC variant, *n* (%)	3 (6.4)	0 (0)	.546
XACT variant, *n* (%)	1 (2.1)	0 (0)	.999
IGFBP variant, *n* (%)	2 (4.3)	0 (0)	.546
FOXO3 variant, *n* (%)	8 (17)	7 (29.2)	.236

Abbreviations: ASCA, anti-*Saccharomyces cerevisiae* antibodies; BMI, body mass index; CARD9, caspase recruitment domain-containing protein 9; CDAI, Crohn’s disease activity index; CRP, C-reactive protein; CS-free CR, corticosteroid-free clinical remission; FC, fecal calprotectin; FOXO3, forkhead box O3; HBI, Harvey–Bradshaw index; IBD, inflammatory bowel disease; IGFBP, insulin-like growth factor-binding protein; IRGM, immunity-related GTPase family M protein; MHC, major histocompatibility complex; NOD2, nucleotide-binding oligomerization domain-containing protein 2; PRO-2, patient-reported outcome 2; SES-CD, simple endoscopic score for Crohn’s disease; SIBDQ, short IBD questionnaire; WBC, white blood cell; XCAT, X-active coating transcript. Values are *n* (%) or median (interquartile range [IQR]).

## Discussion

In this real-world inception cohort of patients with ndCD (all-comers) who were treated and cared for by an MDT in a tertiary center with state-of-the-art management strategies, we showed favorable 1-year outcomes based on the prespecified therapeutic targets. Specifically, sustained CS-free CR reflecting a practical, yet stringent definition that includes no active perianal disease, sustained therapy, and no hospitalization > 3 months was demonstrated in >60% of patients, with similar results between patients with baseline mild–moderate versus moderate–severe disease. Furthermore, we found that a substantial proportion of this cohort achieved all other therapeutic targets such as significant improvement in biomarkers, endoscopic healing, and improved quality of life. In a subset of patients with an uncomplicated phenotype (B1/P0), further improved results were demonstrated, reaching 73% sustained CS-free CR at 1 year. Finally, we showed that uncomplicated phenotype, lower BMI, and lower PRO-2 score at diagnosis could predict the likelihood of reaching sustained CS-free CR at 1 year.

To the best of our knowledge, this is the first study reporting real-world data on outcomes of consecutive, treatment-naïve patients with ndCD treated by an MDT while following contemporary strategies of early intervention, top-down, and tight monitoring. We elected to assess sustained CS-free CR, a strict definition for CR that emulates the desired outcome of interest, especially from the patients’ perspective.

Our results in achieving CS-free CR were similar or better compared with those reports from most registration trials assessing specific therapies, and pivotal clinical trials in the field.^9,10,18–23^ Moreover, most of these trials consider a relatively limited outcome like CDAI < 150 while assessing a specific facet of IBD care—such as drug efficacy, the timing of intervention, or specific management strategies. Finally, almost all RCTs had rigorous inclusion criteria excluding a large proportion of patients with CD,^24,25^ which is also demonstrated in our real-world cohort in which almost two-thirds of trial-ineligible patients (38/60) were actually recommended for advanced therapy.

The favorable results in our study may be partially explained by the characteristics of the study population, patients with early CD, naïve to biologics. Another factor is the rapidity of intervention, about 70% were recommended an advanced treatment strategy to control their disease, of whom 85% started biological therapy within 4 months from diagnosis, similar to contemporary trends observed in the administrative data recently published from the Israeli epi-IIRN.^26^

Additionally, a potential explanation might be the high rate of anti-TNF therapy in this cohort; however, it should be underscored that we had very high rates of compliance and adherence, which might be associated with our structured MDT (>75% assessed and cared for by the IBD-oriented dietitians and the IBD nurse). It is also not unlikely that the close contact of these patients with our ancillary team members who provided guidance, education, and accessibility impacted response and remission rates. We and others have already shown that an MDT approach and improved accessibility to medical consults benefit decision-making, patients’ quality of care, and rates of hospitalizations^27–30^; however, our current results suggest that the MDT approach possibly improves disease outcomes.

We showed that clinical markers of mild disease (uncomplicated phenotype and PRO-2 score) at diagnosis could predict sustained CS-free CR at 1 year. Serology and genetic variants were not associated with outcomes, probably owing to the small sample size and short follow-up period. Nonetheless, smoking, and higher BMI at diagnosis were inversely associated with sustained CS-free CR at 1 year (for each unit increase in BMI, the odds of achieving a favorable outcome would decrease by 1.15 times, or 15%), reflecting the impact of environmental factors on the disease course. This is in line with the recent report by Lopes and colleagues, who demonstrated that adherence to a healthy lifestyle (including nonsmoking and a normal BMI) could prevent CD.^31^ Finally, such aspects of lifestyle can be communicated to patients with ndCD and further addressed by the MDT approach.

This study is not without limitations, it is single-center design, the decision-making process was nonstructured, the cohort is relatively small, and only patients who had assessments at baseline and 1 year were included, all possibly introducing bias. The lack of a control group is another important limitation, especially when attempting to assess the impact of MDT specifically on patient outcomes. In fact, a rigorous RCT evaluation of MDT intervention in patients with IBD is less feasible in practice since the MDT approach in our center has already been incorporated into standard care.

Additionally, the prevalence of anti-TNF therapy was notably high, approximating 60%. This could have been a contributory factor in achieving favorable outcomes. Nonetheless, we posit that this elevated rate of an early advanced therapeutic approach for patients with ndCD aligns with the exigencies of clinical practice in Israel^26,32^ and also the STRIDE-II recommendations.^7^ Moreover, while the utilization rate of anti-TNF therapy may undergo adjustments in certain places due to the introduction of novel pharmacologic agents for the treatment of CD, it will be still pertinent in jurisdictions where economic considerations necessitate the prioritization of biosimilar medications over cost-prohibitive newer therapies.^33^

Finally, there was no 1 specific intervention in this cohort study, but rather a real-world practice utilizing multiple options, mostly tailored, taken together with a specialized MDT. This may appear as a methodological flaw that can imped external validity and generalizability of our findings. However, facing the complexity of CD, this representation of the results is an actual strength. Our study reflects the real-world practice, where we must address the complexities encountered daily, where clinicians manage a diverse patient population and where a universal, curative approach remains elusive.

In conclusion, in this inception cohort of patients with ndCD treated and cared for by an MDT in a real-world setting at a tertiary medical center, there were favorable 1-year outcomes. This cohort represents a mix of patients with ndCD as typically seen in the community and other referral centers with outcomes improved compared to previous reports.^34,35^ It is most likely that the early introduction of effective therapy was the main determinant that impacted therapeutic targets but it is also plausible that the systematic MDT approach, which allowed attention to the multiple facets of CD patients’ care, also had a contributory effect. Therefore, we advocate for the ubiquitous implementation of an MDT approach across all IBD centers/units and in settings where access to a proficient MDT is limited, telemedicine or remote consultations from specialized MDT units that can serve as an effective alternative. This MDT model may allow access to high-quality healthcare and potentially catalyze transformative changes in healthcare delivery systems, thereby enhancing patient care outcomes.

## Supplementary Material

otad064_suppl_Supplementary_Figures_1-2_Tables_1-2

## Data Availability

The data cannot be shared publicly for the privacy of individuals that participated in the study. The data will be shared on reasonable request to the corresponding author with the permission of our local IRB.
