# Supplementary material for: A Real-World Prospective Cohort Study of Patients With Newly Diagnosed Crohn’s Disease Treated by a Multidisciplinary Team: 1-Year Outcomes
Source: Crohns Colitis 360. 2023 Oct 18;5(4):otad064. doi: 10.1093/crocol/otad064 (PMC10629218; doi:10.1093/crocol/otad064)
Supplement: otad064_suppl_Supplementary_Figures_1-2_Tables_1-2 [file otad064_suppl_supplementary_figures_1-2_tables_1-2.docx]

**Supplementary Figure 1:**

**Schematic representation of study design**

**Prospective observation longitudinal study**


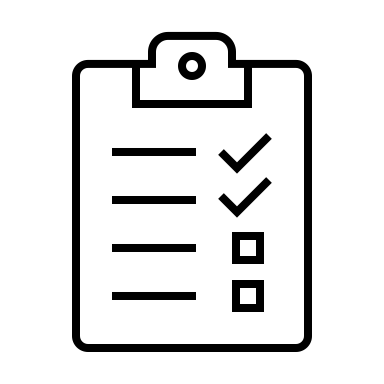


**Patients with a newly diagnosed CD**


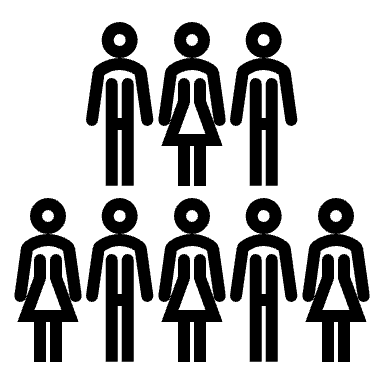


**Enrollment & endoscopy**

**0**

**1**

**Decision making**

**Follow-up visit & endoscopy at one-year**

**Years**


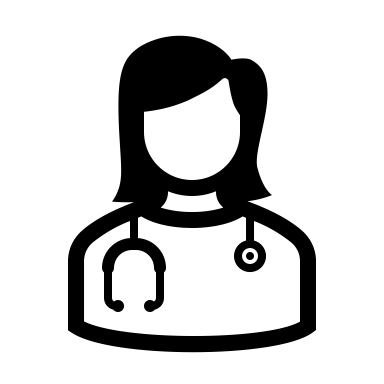

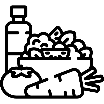

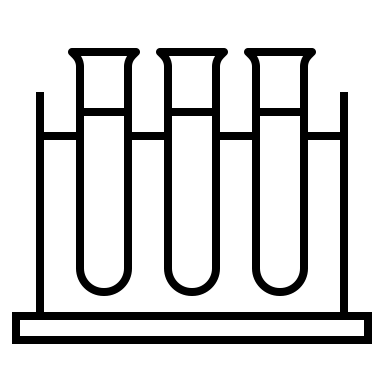


**Multidisciplinary team approach**

**Supplementary Figure 2:**

**Improvement in biomarkers only for patients with elevated CRP or fecal calprotectin at diagnosis**


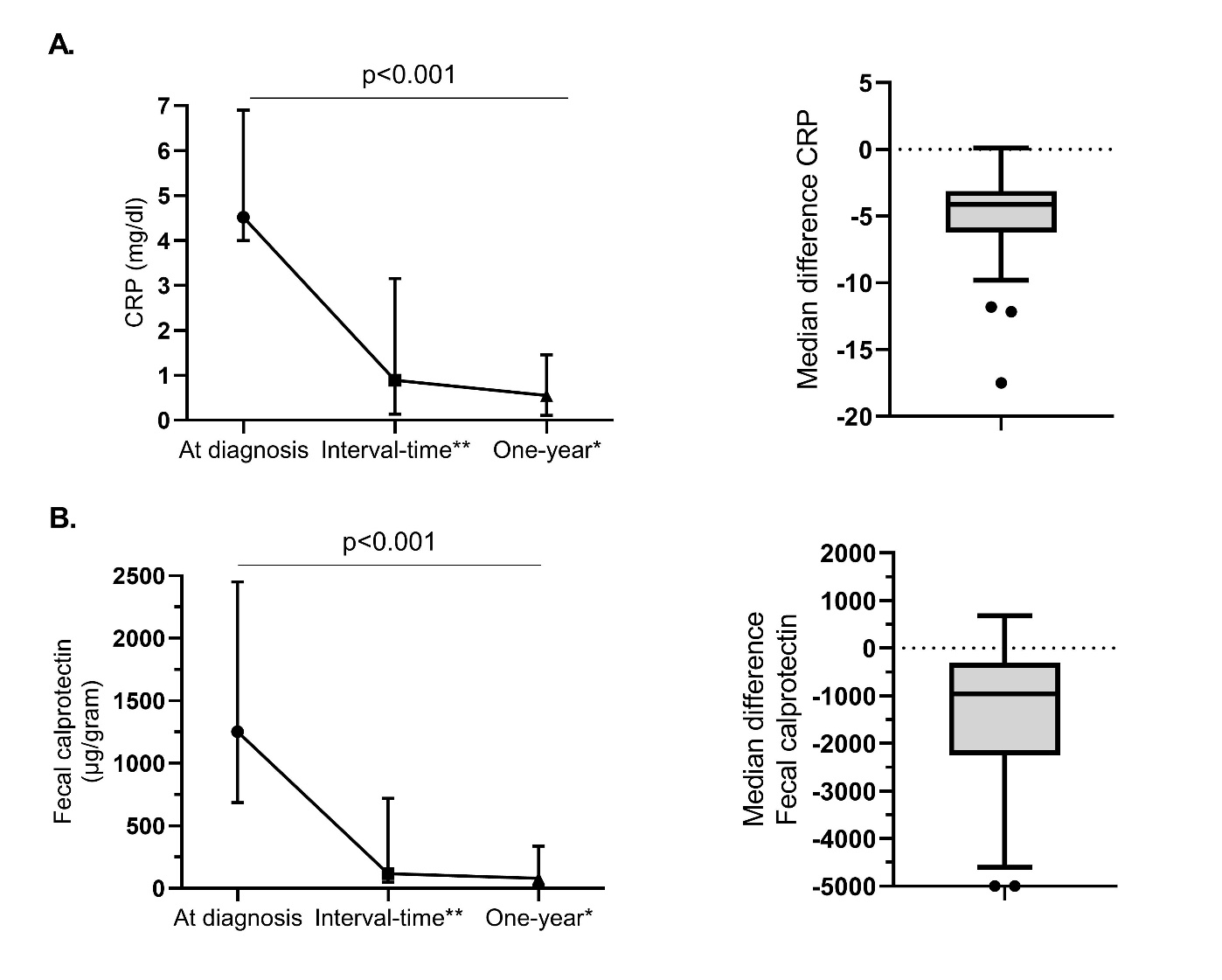


(A) Analysis of a subgroup of patients with elevated CRP >3 mg/dl at diagnosis (n=27).

In this subgroup there was a median decrease from 4.52 mg/dl (IQR 4.00 – 6.90) at diagnosis to 0.55 mg/dl (IQR 0.11 – 1.46) at one-year, p<0.001; median difference was -4.11 mg/dl (IQR [-6.23] – [-3.11]).

(B) Analysis of a subgroup of patients with elevated fecal calprotectin >250 µg/gram at diagnosis (n=49).

In this subgroup there was, there was a median decrease from 1254 µg/gram (IQR 686 – 2450) at diagnosis to 81µg/gram (IQR 19 – 336) at one-year, p<0.001; the median difference was -959 µg/gram (IQR [-2253] – [-305]).

^*^ one-year-latest visit between 9-18 months from diagnosis, **interval-time- the middle of follow up period

**Supplementary Table 1:**

**Susceptible genes variation**

| **Gene name** | **SNP ID** | **Assay ID** |
| --- | --- | --- |
| NOD2 | rs2066847 | C__60383785_10 |
| CARD9 | rs10781499 | C__25957125_10 |
| IRGM | rs11741861 | C_176034574_10 |
| MHC | rs9279411 | See probes below^*^ |
| XACT | rs5929166 | C__29023189_10 |
| IGFBP | rs75764599 | C_100812686_10 |
| FOXO3 | rs147856773 | See probes below^**^ |

^*^MHC_rs9279411

PrimerFW GGGAGCGGAGAGGAGGATTCTGA

PrimerRV TCTTGCCCGGCCAATGCTTATCC

Probe FAM-CCTGCCCGTCCCCC

Probe VIC-CTGCCCCTGTCCCCC

^**^FOXO3_rs147856773

PrimerFW GTGGCATTTTAATGTTCTCAGAACCA

PrimerRV GCCTGAACCTCAATTCCTGATTTC

Probe VIC-CAGTGTGATGTAAGAAA

Probe FAM-TGTGATGTGGTAAGAAA

**Supplementary Table 2:**

**Types and rates of multidisciplinary (MDT) interactions**

| **MDT interactions** | **Number of interactions per patient over one-year, Mean (±SD)** ^*^ |
| --- | --- |
| **Medical visits by the treating physician** | **5.1 (±2.1)** |
| **Dietary consults by an IBD-oriented dietitians** | **1.4 (±1.4)** |
| **Any interactions with the IBD-nurse** | **15.7 (±15.7)** |
| Frontal visits | 5.3 (±4.4) |
| Phone calls | 3.6 (±5.0) |
| E-mail correspondence | 4.4 (±3.9) |
| Text correspondence | 2.4 (±5.9) |

^*^Normalized based on the exact individual follow-up time

**Supplemental methods:**

**Detailed inclusion criteria**

[1] a confirmed new diagnosis of CD according to the ECCO-ESGAR guidelines(16) based on a combination of clinical, biochemical, endoscopic (ileocolonoscopy with biopsies), small bowel assessment with either cross-sectional imaging (computerized tomography [CT] or magnetic resonance imaging [MRI] with enterography protocols [CTE or MRE]), or capsule endoscopy, with typical findings [2] CD diagnosis less than six months before enrollment; [3] naïve to systemic corticosteroids, immunosuppression, or biologic therapy at recruitment (allowed a short-course of budesonide [up to 12 weeks] or oral mesalamine treatment, or antibiotics prior to enrollment) [4] provide a written informed consent.
